# Boosting the efficiency of site-saturation mutagenesis for a difficult-to-randomize gene by a two-step PCR strategy

**DOI:** 10.1007/s00253-018-9041-2

**Published:** 2018-05-21

**Authors:** Aitao Li, Carlos G. Acevedo-Rocha, Manfred T. Reetz

**Affiliations:** 1 Hubei Collaborative Innovation Center for Green Transformation of Bio-resources, Hubei Key Laboratory of Industrial Biotechnology, College of Life Sciences, Hubei University, Wuhan, 430062 China; 2 Max-Planck-Institut für Kohlenforschung, Kaiser-Wilhelm-Platz 1, 45470 Muelheim, Germany; 3 Department of Chemistry, Philipps-Universität, Hans-Meerwein-Strasse 4, 35032 Marburg, Germany; 4 Biosyntia ApS, 2100 Copenhagen, Denmark

**Keywords:** Directed evolution, Site-saturation mutagenesis, Megaprimer, Library quality, P450 monooxygenase, Mutability landscapes

## Abstract

Site-saturation mutagenesis (SSM) has been used in directed evolution of proteins for a long time. As a special form of saturation mutagenesis, it involves individual randomization at a given residue with formation of all 19 amino acids. To date, the most efficient embodiment of SSM is a one-step PCR-based approach using NNK codon degeneracy. However, in the case of difficult-to-randomize genes, SSM may not deliver all of the expected 19 mutants, which compels the user to invest further efforts by applying site-directed mutagenesis for the construction of the missing mutants. To solve this problem, we developed a two-step PCR-based technique in which a mutagenic primer and a non-mutagenic (silent) primer are used to generate a short DNA fragment, which is recovered and then employed as a megaprimer to amplify the whole plasmid. The present two-step and older one-step (partially overlapped primer approach) procedures were compared by utilizing cytochrome P450-BM3, which is a “difficult-to-randomize” gene. The results document the distinct superiority of the new method by checking the library quality on DNA level based on massive sequence data, but also at amino acid level. Various future applications in biotechnology can be expected, including the utilization when constructing mutability landscapes, which provide semi-rational information for identifying hot spots for protein engineering and directed evolution.

**Electronic supplementary material:**

The online version of this article (10.1007/s00253-018-9041-2) contains supplementary material, which is available to authorized users.

## Introduction

**Fig. 1:**
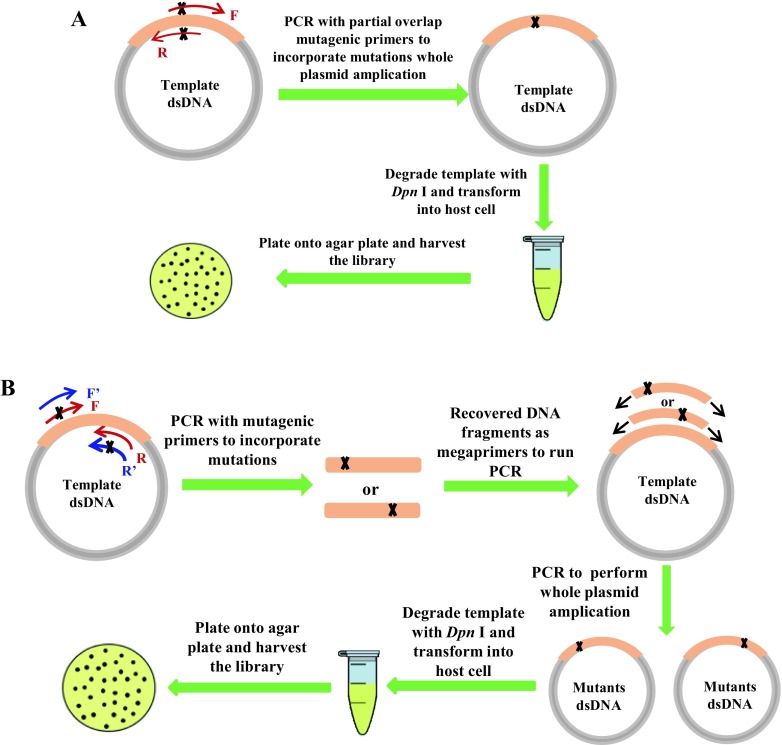
**a** Standard SSM library construction using the one-step PCR approach with partially overlapping primers. A pair of partially overlapping mutagenic primers is used to perform the PCR reaction for incorporating mutations using plasmid pRSFDuet-1 harboring P450-BM3 gene as template; PCR reaction is performed to amplify the whole plasmid, and digestion with *Dpn* I is conducted to eliminate the parent template, followed by transformation into *E*. *coli* BL21 (DE3) and library harvesting. **b** New SSM library construction using the two-step PCR approach with a mutagenic primer and a non-mutagenic (silent) primer. A pair of primers consisting of one forward mutagenic primer and one reverse primer (or one forward primer and one reverse mutagenic prime, depending on the mutational position) is used to perform the PCR reaction for the purpose of incorporating designed mutations using pRSFDuet-1 harboring P450-BM3 gene as template. The amplified short DNA fragments (500 bp in length) containing mutations are recovered and subjected to the second PCR reaction as megaprimers. To amplify the whole plasmid, digestion with *Dpn* I is conducted to eliminate the parent template, followed by transformation into *E*. *coli* BL21 (DE3) and library harvesting

Saturation mutagenesis (SM) is a protein engineering technique that has long been applied in directed evolution of proteins (Wells et al. 1985; Miyazaki and Arnold 1999). SM at a single residue site, which has been called site-saturation mutagenesis (SSM), is widely used to generate all 19 amino acid variants. More recently, SSM has been employed in the construction of mutability landscapes for probing protein sequence–function relationships (Acevedo-Rocha et al. 2018; Hecht et al. 2013; van der Meer et al. 2016a, 2016b). Information-rich sequence–function maps obtained from such mutability landscapes allow researchers to address various problems, including the generation of biomolecular fitness landscapes (Hietpas et al. 2011; Firnberg et al. 2014; Melnikov et al. 2014; Stiffler et al. 2015; Klesmith et al. 2015), therapeutic protein optimization (Whitehead et al. 2012), high-resolution conformational epitope mapping (Kowalsky et al. 2015), and the engineering of protein selectivity (van der Meer et al. 2016a, 2016b) or binding (Park et al. 2015). A variety of different SM and SSM molecular biological methods have been employed during the past two decades (Arndt and Müller 2007; Dominy and Andrews 2003; Georgescu et al. 2003; Hogrefe et al. 2002; Kirsch and Joly 1998; Zheng et al. 2004; Reetz 2004; Wrenbeck et al. 2016; Haller et al. 2016). Currently, the most popular approach is the Stratagene QuikChange^™^ method, which is based on the use of overlapping antiparallel oligonucleotides encoding degenerate codons (Hogrefe et al. 2002). It has proven to be very successful for simple site-directed mutagenesis in so-called rational design of mutant enzymes, but it has some drawbacks when applied to SSM. The PCR reaction is a linear, rather than an exponential amplification process due to the completely overlapped primers (Xia et al. 2015). Since the products from the first cycle cannot serve as templates for subsequent rounds, the PCR amplicon yield is rather low, which means that it is very difficult to obtain the desired number of colonies after transformation (Sullivan et al. 2013). To overcome this problem, different approaches have been developed by using partially overlapped (Zheng et al. 2004) or even non-overlapping primers (Kirsch and Joly 1998), where the resulting amplicon is used as a megaprimer (Sarkar and Sommer 1990; Miyazaki and Takenouchi 2002), thereby completing the plasmid amplification in a second stage PCR. However, these methods fail in the case of recalcitrant targets, especially when cloned in large plasmids, or templates with extensive secondary structures, very AT- or GC-rich base compositions, or long short tandem repeats such as human insulin receptor and kinase (Korbie and Mattick 2008; Sahdev et al. 2007). Other examples of difficult-to-randomize templates include epoxide hydrolase from *Aspergillus niger*, lipases from *Pseudomonas aeruginosa* and *Candida antarctica*, and P450-BM3 from *Bacillus megaterium* (ATCC 14581) (Sanchis et al. 2008), which is in particular a challenging gene of long length (3.3 kb DNA length, GenBank accession number: NZ_CP009920.1) and with very AT-rich (55% in average, but there are regions that exhibit > 60%) base compositions that cannot be randomized easily by traditional QuikChange procedures (Acevedo-Rocha et al. 2015). An improved method of a single two-stage whole-plasmid PCR based on non-overlapping primers was then developed for P450-BM3 genes (Sanchis et al. 2008), but the improved technique is far from perfect for generating high-quality SSM and combinatorial libraries, as shown for BM3 (Hoebenreich et al. 2015). More importantly, in all the above studies, a statistical analysis based on oversampling and massive gene sequencing was not performed, which means that a reliable assessment of library quality at gene level was not possible and certainly not at protein level either. Subsequently, a promising method for creating and evaluating SSM libraries was developed for another “difficult-to-randomize” gene, an Old Yellow Enzyme enoate reductase (Sullivan et al. 2013), based on the Reymond primer design approach (Fig. 1a) (partially overlapped primers region to 25 bp) (Zheng et al. 2004). Nevertheless, a statistical analysis based on oversampling factor of 3 showed that the quality of the created libraries was still not fully satisfactory with a relatively high percentage of parental template (up to 31%). Therefore, a robust and accessible method for the construction of high-quality, user-defined SSM libraries is lacking, especially when recalcitrant gene templates are involved.

In this study, we developed a two-step PCR approach with non-overlapping primers to perform SSM using an enzyme which constitutes a challenge because, as mentioned above, it involves a difficult-to-randomize gene: cytochrome P450-BM3 (Sanchis et al. 2008). According to the new approach, NNK-based library construction is performed with only two primers. This technique is usually more economical than the Trick22c (Kille et al. 2013) and Tang-20c (Tang et al. 2012) strategies, which require more than two primers. Statistically, it is much cheaper to perform NNK mutagenesis, followed by site-directed mutagenesis to obtain the missing mutants (Nov et al. 2013). In the first step of our new strategy, a mutagenic primer and a non-mutagenic (silent) primer are used to perform the first PCR for 28 cycles to obtain short DNA fragments. The recovered DNA fragments then serve as a megaprimer to perform the second PCR for whole plasmid amplification with 24 cycles, and the resulting amplicons are digested with *Dpn* I to eliminate the parental template, followed by transformation into *Escherichia coli* BL21 (DE3) and final library harvesting (Fig. 1b).

For comparison, we also employed the previously best technique based on partially overlapping oligos for the same SSM library construction. The two approaches were systematically analyzed and compared by massive sequencing at different oversampling factors.

## Materials and methods

### Materials

KOD Hot Start DNA Polymerase was obtained from Novagen. Restriction enzyme *Dpn* I was bought from NEB. The oligonucleotides were synthesized by Life Technologies/Thermo Fisher Scientific. Plasmid preparation kit was ordered from Zymo Research, and PCR purification kit was bought from QIAGEN. DNA sequencing was conducted by GATC Biotech. All commercial chemicals were purchased from Sigma-Aldrich, Tokyo Chemical Industry (TCI), or Alfa Aesar. The P450-BM3 gene from *B*. *megaterium* was constructed accordingly, and the sequence is shown in the Supplementary Materials (Fig. S1).

### Site-saturation mutagenesis library construction strategies

The NNK-based SSM libraries were constructed using the one-step PCR approach as well as the two-step PCR approach. For the one-step PCR approach (Fig. 1a), a pair of partially overlapping mutagenic primers is used to perform the PCR reaction; to incorporate mutations using plasmid pRSFDuet-1 harboring P450-BM3 gene as template for whole plasmid amplification, the PCR amplicons were digested with *Dpn* I at 37 °C for 6 h and subjected to transformation and library harvest. For the two-step PCR strategy (Fig. 1b), a pair of primers consisting of one forward mutagenic primer and one non-mutagenic reverse primer (or one non-mutagenic forward primer and one reverse mutagenic prime, which depends on the position of the mutational site) was used to perform the PCR reaction for incorporating designed mutations using pRSFDuet-1 harboring P450-BM3 gene as template. The recovered amplified short DNA fragments (ca. 500 bp in length) as megaprimers were purified and then used in the second PCR reaction to amplify the whole plasmid. Finally, the PCR amplicons were digested with *Dpn* I at 37 °C for 6 h and subjected to transformation and library harvesting.

### PCR amplifications

Based on the method of Reymond (Zheng et al. 2004), the designed degenerate primers containing NNK (forward primer) and MNN (reverse primer) mixtures of bases were used in the one-step PCR approach (Table S1). The primer mixtures containing one forward primer (with NNK codes) and one non-mutagenic reverse primer (or one reverse primer with MNN codes and one non-mutagenic forward primer) were employed in the two-step PCR strategy (Table S1). Each PCR reaction mixture (50 μL) contained 30 μL water, 5 μL KOD hot start polymerase buffer (10×), 3 μL 25 mM MgSO_4_, 5 μL 2 mM dNTPs, 2.5 μL DMSO, 0.5 μL (50~100 ng) template DNA, 100 μM primers mix 0.5 μL each (1 μL 300 ng/μL for megaprimer), and 1 μL KOD hot start polymerase. The PCR conditions for short fragment amplification were as follows: 95 °C 3 min (95 °C 30 s, 56 °C 30 s, 68 °C 40 s) × 28 cycles, 68 °C 120 s, 16 °C 30 min. PCR conditions for amplification of the whole plasmid were as follows: 95 °C 3 min (95 °C 30 s, 60 °C 30 s, 68 °C 5 min 30 s) × 24 cycles, 68 °C 10 min, 16 °C 30 min.

### Library generation and evaluation

After *Dpn* I digestion, the PCR amplicons (2 μL) were directly transformed into 100-μL electrocompetent *E*. *coli* BL21 (DE3). After adding 900-μL SOC medium, the cells were recovered at 37 °C for 1 h and then spread onto two agar plates containing kanamycin (50 μg/mL) with 400- and 600-μL cell cultures, respectively. After incubating for 12–16 h at 37 °C, colonies from one agar plate (600 μL) were collected and re-suspended by adding 1 mL distilled water, followed by plasmid extraction. This “pooled” plasmid sample was then sequenced, and the resulting capillary electropherograms were assessed (Figs. S2–S6) and the Q_pool_ values were calculated to evaluate the degeneracy based on the reported method (Sullivan et al. 2013). Ninety-six individual colonies were picked from another plate and inoculated into 400 μL of LB containing 50 μg/mL kanamycin in a 2-mL 96-well plate. This plate was grown for 12–16 h at 37 °C and 220 rpm; then, 120-μL portions of each saturated culture were transferred to a 0.5-mL 96-well plate containing 50 μL of sterile glycerol (70%, *v*/*v*). Individual plasmid DNA was sequenced by automated rolling circle amplification/fluorescence sequencing protocols which was provided by GATC Biotech.

## Results

### Comparison of library sequencing results

Following the workflows of Fig. 1a, b, SSM libraries using P450-BM3 as template were created by NNK-based randomization at selected single positions Y51, S72, L75, L437, and T438 which line the binding pocket at the Fe-heme domain where catalysis occurs (see the complete sequence of P450 gene in Fig. S1).

We began our study by first testing the partially overlapped primer approach (see primer design in Table S1) to perform the NNK-based SSM experiments at the selected positions. To achieve theoretical 95% library coverage, 96 colonies of on the agar plates (based on oversampling factor of 3) for each individual NNK library were used for sequencing (see sequencing results in Table S2), followed by assessment of quality and diversity. As shown in Table 1, for all of the NNK SSM libraries, a total of 458 complete sequences were obtained, and 22 failed to yield usable sequence data. The number of amino acids identified in each SSM library averaged 16.8 ± 2.2, which corresponds to 84 ± 11% yield in a library sample size of 96 members. At the codon level, an average of 24.8 ± 2.1 codons of the 32 possible were found (corresponding to 77.5 ± 6.5% yield) in a NNK library of 96 members. However, the template contamination is very high, and the fraction of wild-type sequence averaged 41.4 ± 6.6%. All libraries contain > 30% wild-type sequence. The wild-type codon sources are mainly from parent template that survived the *Dpn* I digestion and the wild-type codon present in the NNK primer mixture. Therefore, although it has been shown that in the case of an enoate reductase this method works better than earlier approaches (Sullivan et al. 2013), it performs poorly when turning to the more challenging difficult-to-randomize gene P450-BM3.

**Table 1:** Comparison of one-step and two-step PCR approaches for NNK-based SSM

	Libraries	Number of sequences obtained^a^	Number of amino acids present^b^	Number of codons present^c^	Percent wild type^d^	Qpool value from pooled plasmids^e^
One-step PCR method	Y51X	91	18	25	48	0.30
	S72X	92	15	24	34	0.40
	L75X	91	18	28	35	0.44
	L437X	93	14	22	43	0.59
	T438X	91	19	25	47	0.31
Two-step PCR method	Y51X	91	19	30	4	0.43
	S72X	92	19	28	5	0.47
	L75X	92	19	30	2	0.74
	L437X	93	20	30	5	0.82
	T438X	91	18	28	8	0.67

^a^Number of colonies with complete sequence, which was obtained after sequencing 96 colonies for each library

^b^Number of different amino acids found within the sequence data for a given library (NNK primers theoretically encompass all 20 amino acids with different distributions)

^c^Number of different codes found within the sequence data for a given library (NNK primers theoretically encompass all 32)

^d^Fraction of the sequenced clone that contains the starting codon at the targeted position (assumed to result from wild-type carryover)

^e^Calculated for the entire codon from sequence data obtained from the pooled plasmids isolated after the initial transformation according to the method reported (Sullivan et al. 2013), the weighted average across the three bases of a codon yields a Qpool value between 0 and 1, with 1 indicating perfect randomization

We then turned our attention to the two-step PCR approach according to Fig. 1b, the corresponding five NNK libraries being the goal for comparison purposes. Instead of using partially overlapped primers, a mutagenic primer and a non-mutagenic (silent) primer (Table S1) were used to generate a short DNA fragment, which was recovered and then employed as a megaprimer to amplify the whole plasmid, as reported elsewhere (Sanchis et al. 2008). The assessment of quality and diversity of the libraries is presented in Table 1. In this case, a total of 459 complete sequences were obtained (see sequencing results in Table S3), the number of amino acids identified in each SSM library averaging 19.0 ± 0.7 (corresponding to 95 ± 4% yield), and an average of 29.2 ± 1.1 codons of the 32 possible being found (corresponding to 91.3 ± 3.4% yield). Moreover, the fraction of wild type is only 4.8 ± 2.2%, i.e., all libraries contain < 10% wild type.

These data demonstrate that the present two-step PCR technique is clearly more efficient than the alternative Reymond approach (Zheng et al. 2004) which has previously been shown to be best for other templates. For quality control, we also determined the Q_pool_ value, obtained from the pooled plasmid after initial transformation (see sequencing chromatograms in Figs. S2–S6) as developed by Stewart and coworkers (Sullivan et al. 2013). As a result, much higher Q_pool_ values were achieved from the two-step PCR approach relative to that of the one-step PCR process. Although a Q_pool_ value of > 0.70 has been proposed as threshold to obtain a representative number of samples (Sullivan et al. 2013), in several cases, we obtained almost all 19 mutants even when Q_pool_ values vary from 0.31 to 0.82 in the present study (Table 1) and from 0.48 to 0.55 elsewhere (Acevedo-Rocha et al. 2015). For this reason, the Q_pool_ value is a good measure of effective randomization, but it does not guarantee the creation of all target variants. In addition, the number of colonies developed on the agar plates based on 200-μL cell culture was also compared (Fig. S7). An average of ~ 1000 colonies were formed from the two-PCR step approach, which is distinctly higher than that of the one-PCR step approach (average of only ~ 200 colonies), meaning that more usable fragments are formed with the two-step PCR approach. This demonstrates once more the superiority of the non-overlapping two-step PCR method.

### Assessing library quality in terms of genetic and residue diversity

**Fig. 2:**
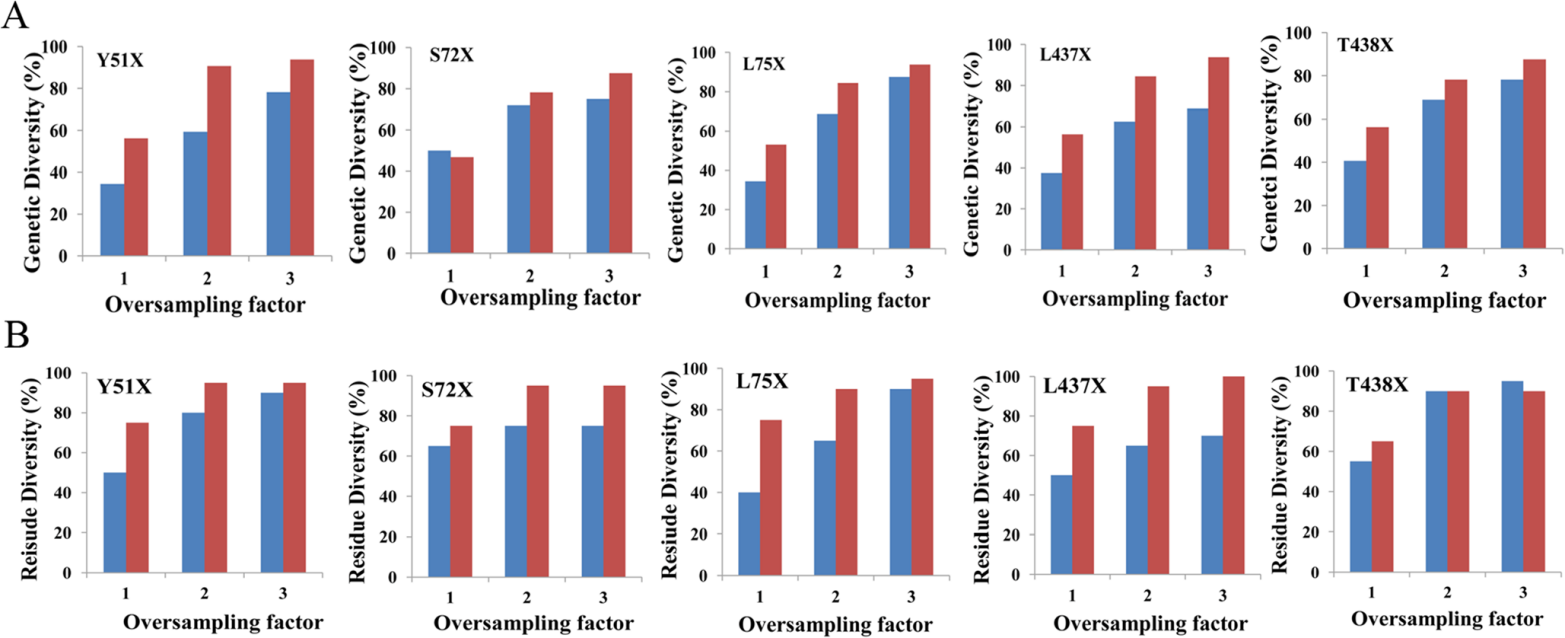
Genetic diversity (**a**) and residue diversity (**b**) for each NNK-based SSM library for both one-step PCR (blue bar) and two-step (red bar) PCR methods based on different oversampling factors. Genetic diversity is defined by the ratio of the number of codons obtained after sequencing to the number of theoretically possible codons expected (32) expressed in percentage; residue diversity is defined by the ratio of the number of residues obtained after sequencing to the number of theoretically possible residues expected (20) expressed in percentage

The comparison of genetic diversity and residue diversity based on oversampling factors from 1 to 3 for individual SSM libraries was also conducted. As shown in Fig. 2, both genetic and residue diversity of the SSM libraries generated with the two-step PCR method are much better than the one-step PCR method. More importantly, when using the two-step PCR method, residue diversity also reached the maximum value with an oversampling factor of 2 in some cases of libraries (Fig. 2b), indicating that oversampling by a factor of 3 may not be necessary.

### Assessing library quality in terms of residue distribution

**Fig. 3:**
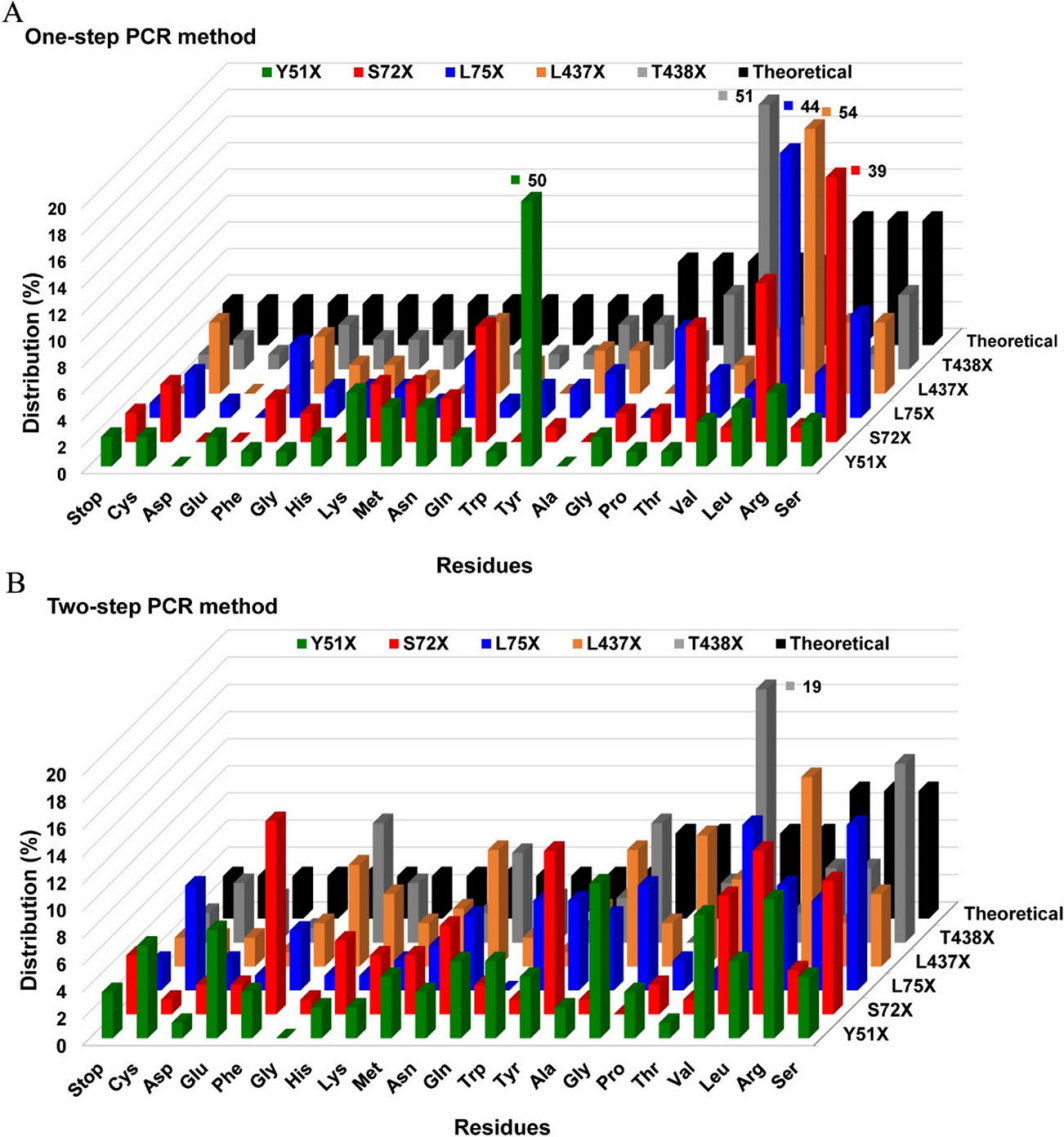
Residue distribution (expressed in percentage) at each NNK-based SSM library for both one-step PCR (**a**) and two-step PCR (**b**) methods based on oversampling factor of 3. The theoretical distribution (expected distribution value) for each residue in a given NNK-based SSM library is shown with black bar. In some cases which exceed the scope are shown with Arabic numerals

The residue distribution for individual SSM library was further analyzed for both one-step and two-step PCR methods based on oversampling factor of 3. As shown in Fig. 3, high degree of parental amino acid bias is present in the one-step method with the distribution values ranging from 39 to 51% (Fig. 3a), while parental amino acid distribution values were greatly reduced in the two-step method (5–19% distribution for wild type) (Fig. 3b). In addition, overall, fewer residues were missed in the libraries created with the two-step vs. one-step PCR method. In library Y51X, two (Asp, Ala) and one (His) residues were missed for the one- and two-step PCR method, respectively (Fig. S8A). In library S72X, five (Asp, Glu, Ile, Trp, Ala) residues were missed with the one-PCR method, while only one (Gly) could not be obtained by the improved method (Fig. S8B). In the case of library L75X, two residues (Glu, Gly) were not generated using the old method and only one (Gln) was not sampled with the new method (Fig. S8C). Most significantly, the two-step method generated all 19 mutants, whereas the one-step method failed to provide six residues (Asp, Glu, Met, Trp, Gly, Pro) in library L437X (Fig. S8D). Lastly, one amino acid (Glu) was missed by the one-step PCR method, while two residues (Phe, Gly) were not sampled using the two-step PCR method in library T438 (Fig. S8E).

### Statistical analysis for all SSM libraries at both codon and residue levels

**Fig. 4:**
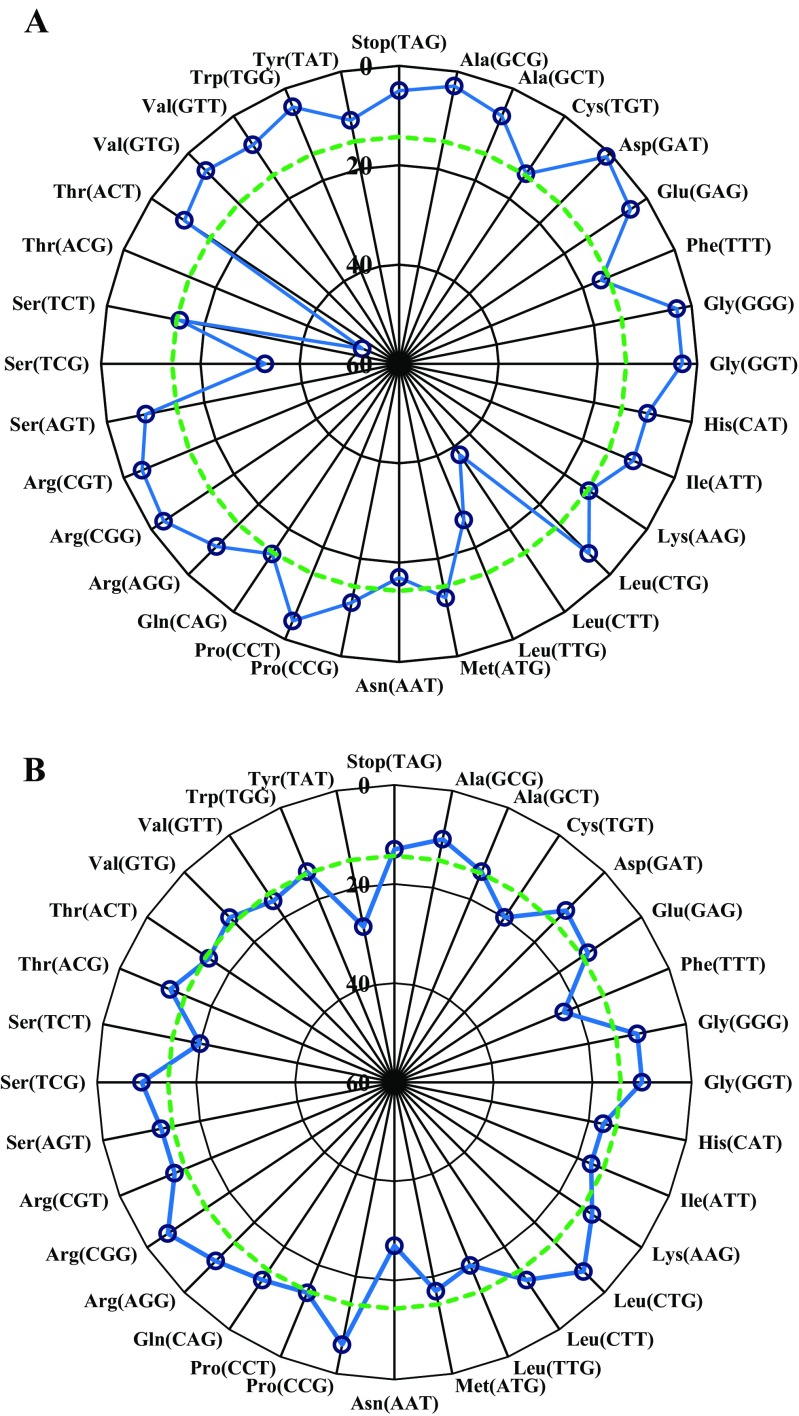
Codon distributions across all NNK-based SSM libraries for both one-step PCR (**a**) and two-step PCR (**b**) methods. The aggregate number of occurrences of each codon in all five of the SSM libraries is listed with a maximum of 60 in the center of the plot. This scale was chosen based on the number of occurrences of the most commonly observed codon (ACG) in the one-step PCR approach library. The dashed green line represents the expected aggregate occurrence of each codon based on perfectly random replacements at each position. Codons present from wild-type carryover have been eliminated from the data for libraries of Y51X and L437X, since the wild-type codes in both libraries cannot be generated with NNK/MNN

Next, at both codon and residue levels, individual codons and identified amino acids were statistically analyzed and compared in all SSM libraries (458 complete sequences for one-step method and 459 complete sequences for two-step method). For the one-step PCR method (Fig. 4), most codons deviated from the expected frequency based on random base replacement. Among the 32 codons, most of them are poorly represented (lower frequency than the expected values), the highest frequency codon proved to be ACG (Thr) with a total number of 52 and to lesser extent codon of CTT (with a total number of 38). This was mainly caused by the wild-type carryover from parent template survival and wild-type codon present in the NNK primer. In contrast, for the SSM libraries constructed by the non-overlapping two-step PCR method, as shown in Fig. 4b, most codons are near the expected frequency (Fig. 4b), with only a slight deviation in some cases, such as TAT (Tyr) and AAT (Asn).

**Fig. 5:**
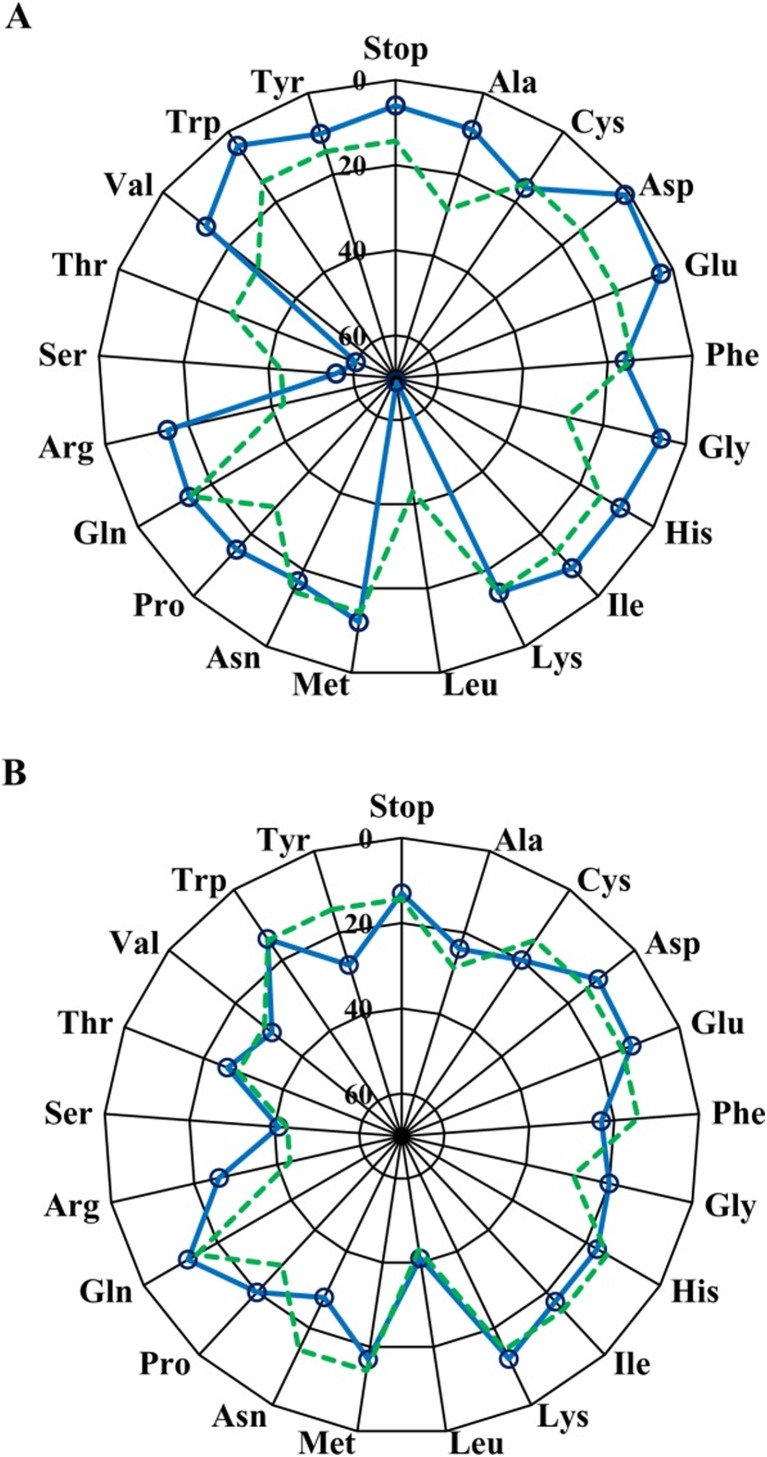
Residue distributions across all NNK-based SSM libraries for both one-step PCR technique (**a**) and two-step PCR approach (**b**). The aggregate number of occurrences of each amino acid in all five of the saturation libraries is listed with a maximum of 70 in the center of the plot. This scale was chosen based on the number of occurrences of the most commonly observed amino acid (Leu) in the libraries constructed by the one-step PCR approach. The expected aggregate occurrence of each amino acid based on perfectly random replacements at each position is also shown (green dashed lines)

Finally, the comparison of amino acid distributions across all SSM libraries between the two methods was conducted (Fig. 5a, b). Different from the codon distribution, residues are not expected in equimolar amounts, since an NNK mixture contains multiple codons for some amino acids. However, a trend similar to the analysis at codon level was obtained: Most residues deviated from the expected frequency in the SSM library generated by the one-step PCR method, especially for the residues Thr and Leu. In contrast, for the improved method, most residues closely match the predicted level. This type of analysis constitutes yet another way to demonstrate the superiority of the two-step PCR approach with non-overlapping primers.

## Discussion

In this study, we have developed a two-step PCR method for constructing NNK-based SSM libraries and compared the results with the performance of the conventional approach based on partially overlapped primers in a one-step PCR process, the difficult-to-randomize P450-BM3 gene template serving as the model system. This recalcitrant system was specifically chosen in order to test our strategy in difficult situations. The two-step PCR-based technique is based on the use of a mutagenic primer and a non-mutagenic (silent) primer with generation of a short DNA fragment. The latter is recovered and then employed as a megaprimer to amplify the whole plasmid. In order to assess and compare both approaches, several statistical techniques were employed. Distinctly higher efficiency of the two-step approach was demonstrated by statistical analysis based on oversampling factor of 3 at both codon and amino acid levels. Moreover, the two-step PCR technique showed much better library quality in terms of reduced wild-type contamination and in approaching nearly ideal codon and residue distributions. The difference in efficacy has practical and economical significance. When generating an incomplete set of single mutants using the one-step PCR approach, the user needs to perform site-specific mutagenesis for obtaining the missing mutants, which means additional work and expenses (Acevedo-Rocha et al. 2015).

In summary, the method described herein provides a superior means to create SSM libraries at single residue sites, thus improving the ability to engineer enzymes on a semi-rational basis. In particular, it can be expected to be viable when constructing mutability landscapes (Acevedo-Rocha et al. 2018; Hecht et al. 2013; van der Meer et al. 2016a, 2016b) in the quest to identify all “hotspots” at any place in the amino acid sequence of an enzyme in a reliable manner. This kind of information can be exploited subsequently for combinatorial mutagenesis with generation of superior enzymes having desired properties. Finally, we point out that the term “quality of a mutant library” has been used many times in directed evolution studies, generally on protein level referring to such enzyme properties as activity, selectivity, or stability. However, this constitutes a restricted view. In contrast, the statistical techniques of the type described herein for actually assessing quality on DNA and subsequently also on amino acid level provide a complete picture and enable the researcher to draw relevant conclusions.

## Electronic supplementary material

ESM 1 (PDF 742 kb)
