# Supplementary material for: Boosting the efficiency of site-saturation mutagenesis for a difficult-to-randomize gene by a two-step PCR strategy
Source: Appl Microbiol Biotechnol. 2018 May 21;102(14):6095–103. doi: 10.1007/s00253-018-9041-2 (PMC6013526; doi:10.1007/s00253-018-9041-2)
Supplement: Supplementary file 1 — (PDF 742 kb) [file 253_2018_9041_MOESM1_ESM.pdf]

Supplementary Materials

Applied Microbiology and Biotechnology

Boosting the Efficiency of Site-Saturation Mutagenesis for a Difficult-to-Randomize Gene by a Two-Step PCR Strategy

Aitao Li,[a,b,c] Carlos G. Acevedo-Rocha,[d] and Manfred T. Reetz [b,c]*

[a]Hubei Collaborative Innovation Center for Green Transformation of Bio-resources, Hubei Key Laboratory of Industrial Biotechnology, College of Life Sciences, Hubei University, 430062, Wuhan, China.

[b]Max-Planck-Institut für Kohlenforschung, Kaiser-Wilhelm-Platz 1, 45470 Muelheim, Germany

[c]Department of Chemistry, Philipps-University, Hans-Meerwein-Strasse 4, 35032 Marburg, Germany

[d]Biosyntia ApS, Copenhagen, 2100 Denmark

Corresponding author

Manfred T. Reetz

Phone: +49 6421 28 25500

Email: reetz@mpi-muelheim.mpg.de

ORCID

Aitao Li: 0000-0001-6036-4058

Manfred T. Reetz：0000-0001-6246-647X

Legends of figures and tables

Fig. S1. The plasmid pRSFduet-1 harboring P450-BM3 gene with a total length of 3222 bp. The heme domain sequence of P450-BM3 is underlined, the target positions are bold-marked.

Fig. S2. Sequencing chromatograms for NNK-based library Y51X generated from one-step PCR or two-step PCR processes.

Fig. S3. Sequencing chromatograms for NNK-based library S72X generated from one-step PCR or two-step PCR processes.

Fig. S4. Sequencing chromatograms for NNK-based library L75X generated from one-step PCR or two-step PCR processes.

Fig. S5. Sequencing chromatograms for NNK-based library L437X generated from one-step PCR or two-step PCR processes.

Fig. S6. Sequencing chromatograms for NNK-based library T438X generated from one-step PCR or two-step PCR processes.

Fig. S7. Comparison of number of colonies developed on the agar plates for different NNK-based SSM libraries. (A) SSM libraries constructed by partially over-lapped primer in one-step PCR process; (B) SSM libraries constructed by non-overlapping primer in two-step PCR process.

Fig. S8. Comparison of amino acid distribution for different positions (Y51, S72, L75, L437 and T438) for both one-step PCR and two-step PCR methods.

Table S1. Sequence of primers used in this study.

Table S2. Screening results of one-step PCR approach with partially overlapped primer for NNK-based site-saturation mutagenesis.

Table S3. Screening results of two-step PCR approach with non-overlapping primers for NNK-based site-saturation mutagenesis.

ATGAAACATCACCATCACCATCACCCCATGAGCGATTACGACATCCCCACTACTGAGAAT
CTTTATTTTCAGGGCGCAATTAAAGAAATGCCTCAGCCAAAAACGTTTGGAGAGCTTAAA
AATTTACCGTTATTAAACACAGATAAACCGGTTCAAGCTTTGATGAAAATTGCGGATGAA
TTAGGAGAAATCTTTAAATTCGAGGCGCCTGGTTGGGTAACGCGC***TAC***TTATCAAGTCAGC
GTCTAATTAAAGAAGCATGCGATGAATCACGCTTTGATAAAAACTTA***TCG***CAAGCG***CTT***A
AATACCTAAGAGAATTCCTAGGAGACGGGTTAGGATTAAGCTGGACGCATGAAAAAAATT
GGAAAAAAGCGCATAATATCTTACTTCCAAGCTTCAGTCAGCAGGCAATGAAAGGCTATC
ATGCGATGATGGTCGATATCGCCGTGCAGCTTGTTCAAAAGTGGGAGCGTCTAAATGCAG
ATGAGCATATTGAAGTACCGGAAGACATGACACGTTTAACGCTTGATACAATTGGTCTTTG
CGGCTTTAACTATCGCTTTAACAGCTTTTACCGAGATCAGCCTCATCCATTTATTACAAGT
ATGGTCCGTGCACTGGATGAAGCAATGAACAAGCTGCAGCGAGCAAATCCAGACGACCC
AGCTTATGATGAAAACAAGCGCCAGTTTCAAGAAGATATCAAGGTGATGAACGACCTAGT
AGATAAAATTATTGCAGATCGCAAAGCAAGCGGTGAACAAAGCGATGATTTATTAACGCA
TATGCTAAACGGAAAAGATCCAGAAACGGGTGAGCCGCTTGATGACGAGAACATTCGCTA
TCAAATTATTACATTCTTAATTGCGGGACACGAAACAACAAGTGGTCTTTTATCATTTGCG
CTGTATTTCTTAGTGAAAAATCCACATGTATTACAAAAAGCAGCAGAAGAAGCAGCACGA
GTTCTAGTAGATCCTGTTCCAAGCTACAAACAAGTCAAACAGCTTAAATATGTCGGCATG
GTCTTAAACGAAGCGCTGCGCTTATGGCCAACTGCTCCTGCGTTTTCCCTATATGCAAAAG
AAGATACGGTGCTTGGAGGAGAATATCCTTTAGAAAAAGGCGACGAACTAATGGTTCTGA
TTCCTCAGCTTCACCGTGATAAAACAATTTGGGGAGACGATGTGGAAGAGTTCCGTCCAG
AGCGTTTTGAAAATCCAAGTGCGATTCCGCAGCATGCGTTTAAACCGTTTGGAAACGGTC
AGCGTGCGTGTATCGGTCAGCAGTTCGCTCTTCATGAAGCAACGCTGGTACTTGGTATGAT
GCTAAAACACTTTGACTTTGAAGATCATACAAACTACGAGCTGGATATTAAAGAAACT***TT***
AACGTTAAAACCTGAAGGCTTTGTGGTAAAAGCAAAATCGAAAAAAATTCCGCTTGGCGG
TATTCCTTCACCTAGCACTGAACAGTCTGCTAAAAAAGTACGCAAAAAGGCAGAAAACGC
TCATAATACGCCGCTGCTTGTGCTATACGGTTCAAATATGGGAACAGCTGAAGGAACGGC
GCGTGATTTAGCAGATATTGCAATGAGCAAAGGATTTGCACCGCAGGTCGCAACGCTTGA
TTCACACGCCGGAAATCTTCCGCGCGAAGGAGCTGTATTAATTGTAACGGCGTCTTATAAC
GGTCATCCGCCTGATAACGCAAAGCAATTTGTCGACTGGTTAGACCAAGCGTCTGCTGAT
GAAGTAAAAGGCGTTCGCTACTCCGTATTTGGATGCGGCGATAAAAACTGGGCTACTACG
TATCAAAAAGTGCCTGCTTTTATCGATGAAACGCTTGCCGCTAAAGGGGCAGAAAACATC
GCTGACCGCGGTGAAGCAGATGCAAGCGACGACTTTGAAGGCACATATGAAGAATGGCG
TGAACATATGTGGAGTGACGTAGCAGCCTACTTTAACCTCGACATTGAAAACAGTGAAGA
TAATAAATCTACTCTTTCACTTCAATTTGTCGACAGCGCCGCGGATATGCCGCTTGCGAAA
ATGCACGGTGCGTTTTCAACGAACGTCGTAGCAAGCAAAGAACTTCAACAGCCAGGCAGT
GCACGAAGCACGCGACATCTTGAAATTGAACTTCCAAAAGAAGCTTCTTATCAAGAAGGA
GATCATTTAGGTGTTATTCCTCGCAACTATGAAGGAATAGTAAACCGTGTAACAGCAAGG
TTCGGCCTAGATGCATCACAGCAAATCCGTCTGGAAGCAGAAGAAGAAAAATTAGCTCAT
TTGCCACTCGCTAAAACAGTATCCGTAGAAGAGCTTCTGCAATACGTGGAGCTTCAAGAT
CCTGTTACGCGCACGCAGCTTCGCGCAATGGCTGCTAAAACGGTCTGCCCGCCGCATAAA
GTAGAGCTTGAAGCCTTGCTTGAAAAGCAAGCCTACAAAGAACAAGTGCTGGCAAAACGT
TTAACAATGCTTGAACTGCTTGAAAAATACCCGGCGTGTGAAATGAAATTCAGCGAATTT
ATCGCCCTTCTGCCAAGCATACGCCCGCGCTATTACTCGATTTCTTCATCACCTCGTGTCGA
TGAAAAACAAGCAAGCATCACGGTCAGCGTTGTCTCAGGAGAAGCGTGGAGCGGATATG
GAGAATATAAAGGAATTGCGTCGAACTATCTTGCCGAGCTGCAAGAAGGAGATACGATTA
CGTGCTTTATTTCCACACCGCAGTCAGAATTTACGCTGCCAAAAGACCCTGAAACGCCGCT
TATCATGGTCGGACCGGGAACAGGCGTCGCGCCGTTTAGAGGCTTTGTGCAGGCGCGCAA
ACAGCTAAAAGAACAAGGACAGTCACTTGGAGAAGCACATTTATACTTCGGCTGCCGTTC
ACCTCATGAAGACTATCTGTATCAAGAAGAGCTTGAAAACGCCCAAAGCGAAGGCATCAT
TACGCTTCATACCGCTTTTTCTCGCATGCCAAATCAGCCGAAAACATACGTTCAGCACGTA
ATGGAACAAGACGGCAAGAAATTGATTGAACTTCTTGATCAAGGAGCGCACTTCTATATT
TGCGGAGACGGAAGCCAAATGGCACCTGCCGTTGAAGCAACGCTTATGAAAAGCTATGCT
GACGTTCACCAAGTGAGTGAAGCAGACGCTCGCTTATGGCTGCAGCAGCTAGAAGAAAAA
GGCCGATACGCAAAAGACGTGTGGGCTGGGTAA

Fig. S1. The plasmid pRSFduet-1 harboring P450-BM3 gene (GenBank accession number: NZ_CP009920.1) with a total length of 3222 bp. The heme domain sequence of P450-BM3 is underlined, the target positions are bold-marked.

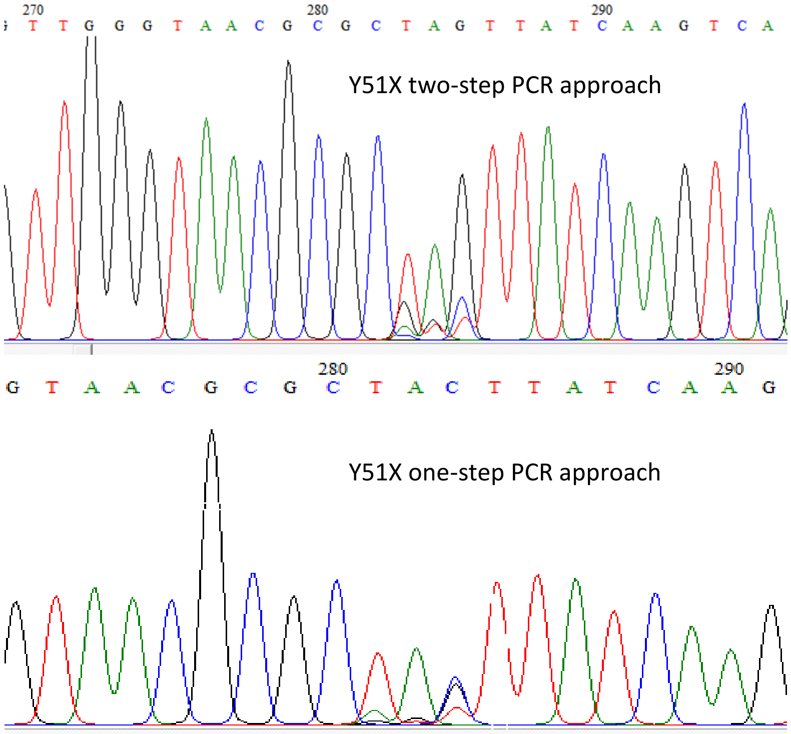

Fig. S2. Sequencing chromatograms for NNK-based library Y51X generated from one-step PCR or two-step PCR processes.

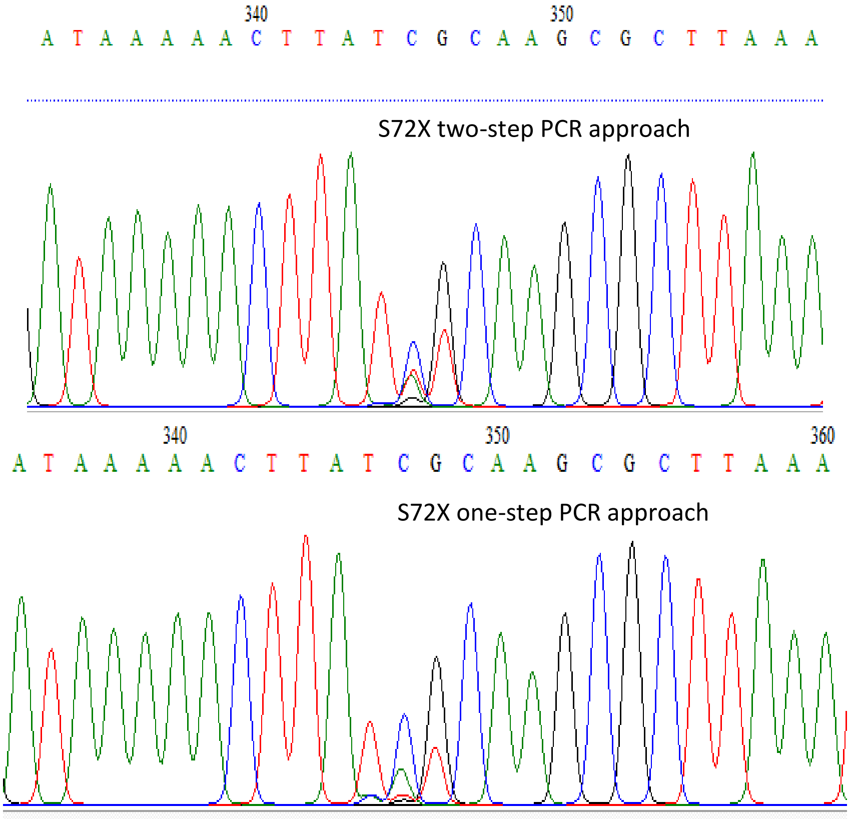

Fig. S3. Sequencing chromatograms for NNK-based library S72X generated from one-step PCR or two-step PCR processes.

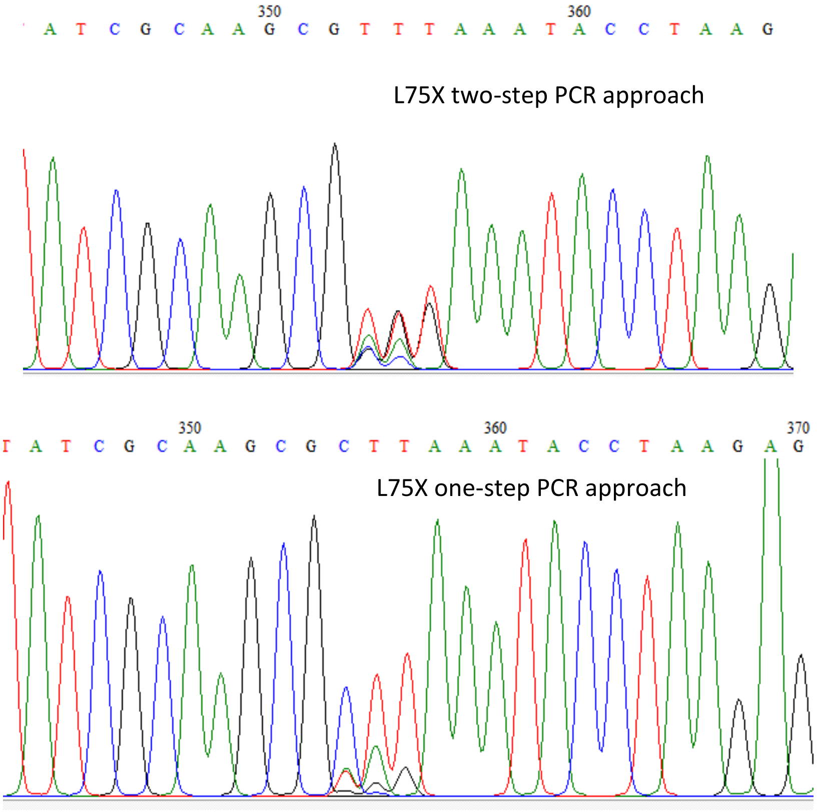

Fig. S4. Sequencing chromatograms for NNK-based library L75X generated from one-step PCR or two-step PCR processes.

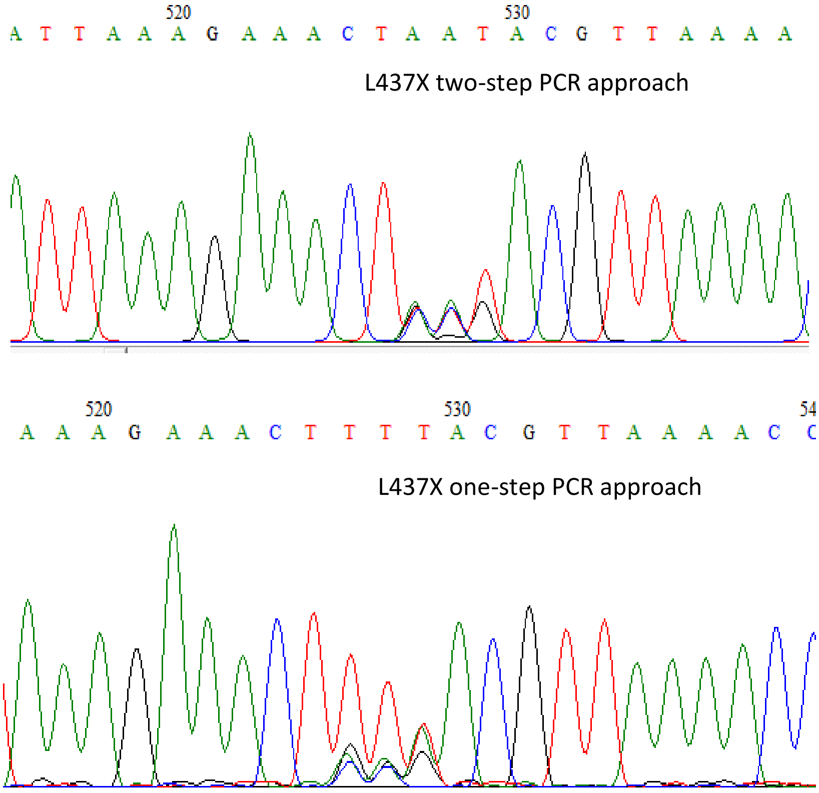

Fig. S5. Sequencing chromatograms for NNK-based library L437X generated from one-step PCR or two-step PCR processes.

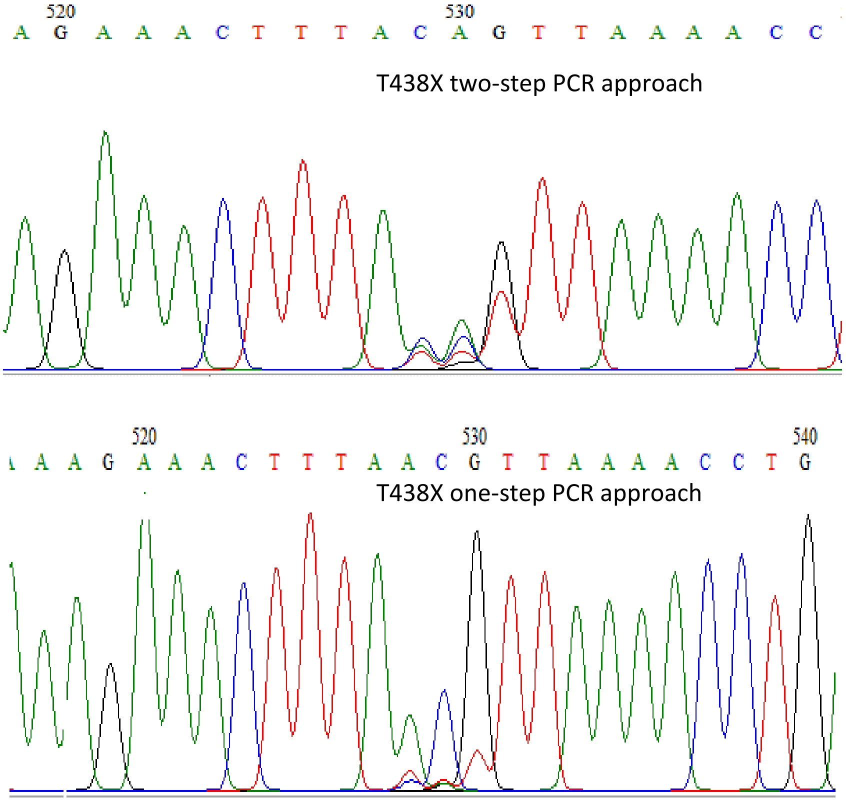

Fig. S6. Sequencing chromatograms for NNK-based library T438X generated from one-step PCR or two-step PCR processes.

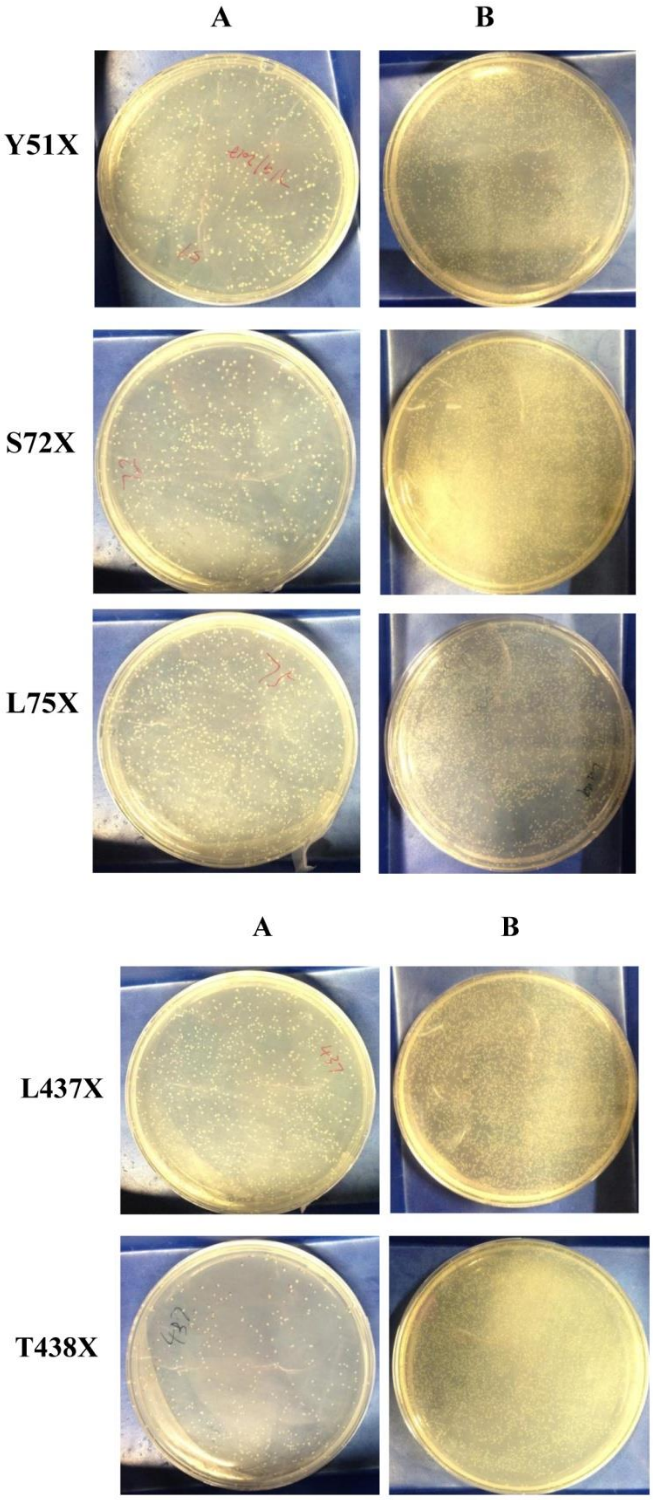

Fig. S7. Comparison of number of colonies developed on the agar plates for different NNK-based SSM libraries. (A) SSM libraries constructed by partially over-lapped primer in one-step PCR process; (B) SSM libraries constructed by non-overlapping primer in two-step PCR process.

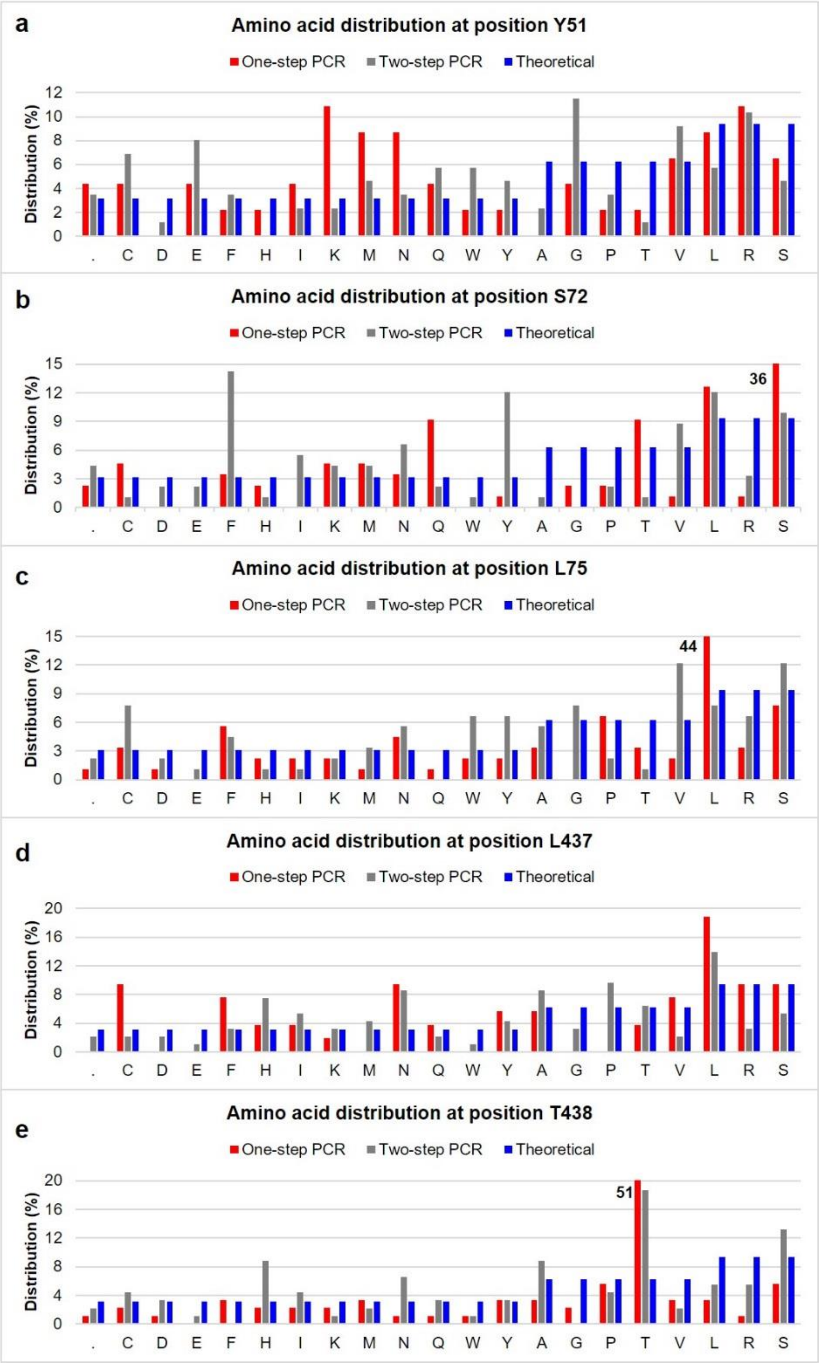

Fig. S8. Comparison of amino acid distribution for different positions (Y51, S72, L75, L437 and T438) for both one-step PCR and two-step PCR methods.

Table S1. Sequence of primers used in this study.

	libraries	Wild-type code	Primer	Primer sequence from 5’ to 3’
One-step PCR	Y51X	TAC	Forward	GGGTAACGCGC**NNK**TTATCAAGTCAGCGTCTAATTAAAG
			Reverse	TGACTTGATAA**MNN**GCGCGTTACCCAACCAGGCGCCTCG
	S72X	TCG	Forward	GATAAAAACTTA**NNK**CAAGCGCTTAAATACCTAAGAGAATTC
			Reverse	TTAAGCGCTTG**MNN**TAAGTTTTTATCAAAGCGTGATTCATC
	L75X	CTT	Forward	CTTATCGCAAGCG**NNK**AAATACCTAAGAGAATTCCTAGG
			Reverse	CTTAGGTATTT**MNN**CGCTTGCGATAAGTTTTTATCAAAGCG
	L437X	TTA	Forward	TTAAAGAAACT**NNK**ACGTTAAAACCTGAAGGCTTTGTGG
			Reverse	CAGGTTTTAACGT**MNN**AGTTTCTTTAATATCCAGCTCGTAG
	T438X	ACG	Forward	AAGAAACTTTA**NNK**TTAAAACCTGAAGGCTTTGTGGTAAAAG
			Reverse	CAGGTTTTAA**MNN**TAAAGTTTCTTTAATATCCAGCTCGTAG
Two-step PCR	Y51X	TAC	Forward	GGGTAACGCGC**NNK**TTATCAAGTCAGCGTCTAATTAAAG
			Reverse[b]	CATCGCTTTGTTCACCGCTTGCTTTGCGATCTGC
	S72X	TCG	Forward	GATAAAAACTTA**NNK**CAAGCGCTTAAATACCTAAGAGAATTC
			Reverse[b]	CATCGCTTTGTTCACCGCTTGCTTTGCGATCTGC
	L75X	CTT	Forward	CTTATCGCAAGCG**NNK**AAATACCTAAGAGAATTCCTAGG
			Reverse[b]	CATCGCTTTGTTCACCGCTTGCTTTGCGATCTGC
	L437X	TTA	Forward[c]	GCAGATCGCAAAGCAAGCGGTGAACAAAGCGATG
			Reverse	CAGGTTTTAACGT**MNN**AGTTTCTTTAATATCCAGCTCGTAG
	T438X	ACG	Forward[c]	GCAGATCGCAAAGCAAGCGGTGAACAAAGCGATG
			Reverse	CAGGTTTTAA**MNN**TAAAGTTTCTTTAATATCCAGCTCGTAG

Bold-marked bases in the primers indicate positions that contain the mutagenic bases in the primer sequence.

[a] N = A, C, G, T (equimolar amounts); K = G, T (equimolar amounts); M = A, C (equimolar amounts)

[b] The same non-mutagenic reverse primer, which was shared for construction of libraries of Y51X, S72X and L75X.

[c] The same non-mutagenic forward primer (reverse and complementary form of the primer mentioned in footnote b), which was shared for libraries construction of L72X and T438X.

Table S2. Screening results of one-step PCR approach with partially overlapped primer for NNK-based site-saturation mutagenesis.

	Y51X		S72X		L75X		L437X		T438X
1	AAG	1	AAG	1	AAG	1	AAG	1	AAG
2	AAG	2	AAG	2	AAG	2	AAT	2	AAG
3	AAG	3	AAG	3	AAT	3	AAT	3	AAT
4	AAG	4	AAG	4	AAT	4	AAT	4	ACG
5	AAG	5	AAT	5	AAT	5	AAT	5	ACG
6	AAT	6	AAT	6	AAT	6	AAT	6	ACG
7	AAT	7	AAT	7	ACG	7	ACG	7	ACG
8	AAT	8	ACG	8	ACG	8	ACG	8	ACG
9	AAT	9	ACG	9	ACT	9	AGG	9	ACG
10	ACG	10	ACG	10	AGG	10	AGG	10	ACG
11	AGG	11	ACG	11	AGT	11	AGT	11	ACG
12	AGG	12	ACT	12	ATG	12	AGT	12	ACG
13	AGG	13	ACT	13	ATT	13	ATT	13	ACG
14	AGT	14	ACT	14	ATT	14	ATT	14	ACG
15	ATG	15	ACT	15	CAG	15	CAG	15	ACG
16	ATG	16	AGG	16	CAT	16	CAG	16	ACG
17	ATG	17	AGT	17	CAT	17	CAT	17	ACG
18	ATG	18	AGT	18	CCG	18	CAT	18	ACG
19	ATT	19	ATG	19	CCG	19	CGG	19	ACG
20	ATT	20	ATG	20	CCG	20	CGG	20	ACG
21	ATT	21	ATG	21	CCG	21	CGT	21	ACG
22	CAG	22	ATG	22	CCT	22	CTG	22	ACG
23	CAG	23	CAG	23	CCT	23	CTT	23	ACG
24	CAT	24	CAG	24	CGG	24	CTT	24	ACG
25	CCT	25	CAG	25	CGT	25	GCT	25	ACG
26	CGT	26	CAG	26	CTG	26	GCT	26	ACG
27	CGT	27	CAG	27	CTG	27	GCT	27	ACG
28	CTT	28	CAG	28	CTT	28	GTG	28	ACG
29	GAG	29	CAG	29	CTT	29	GTT	29	ACG
30	GAG	30	CAG	30	CTT	30	GTT	30	ACG
31	GGT	31	CAT	31	CTT	31	GTT	31	ACG
32	GGT	32	CAT	32	CTT	32	TAT	32	ACG
33	GTG	33	CCG	33	CTT	33	TAT	33	ACG
34	GTG	34	CCT	34	CTT	34	TAT	34	ACG
35	GTT	35	CTG	35	CTT	35	TCG	35	ACG
36	TAC	36	CTG	36	CTT	36	TCT	36	ACG
37	TAC	37	CTT	37	CTT	37	TCT	37	ACG
38	TAC	38	CTT	38	CTT	38	TGT	38	ACG
39	TAC	39	CTT	39	CTT	39	TGT	39	ACG
40	TAC	40	GAG	40	CTT	40	TGT	40	ACG
41	TAC	41	GGG	41	CTT	41	TGT	41	ACG
42	TAC	42	GGT	42	CTT	42	TGT	42	ACG
43	TAC	43	TAG	43	CTT	43	TTA	43	ACG
44	TAC	44	TAG	44	CTT	44	TTA	44	ACG
45	TAC	45	TAT	45	CTT	45	TTA	45	ACG

46	TAC	46	TCG	46	CTT	46	TTA	46	ACG
47	TAC	47	TCG	47	CTT	47	TTA	47	ACT
48	TAC	48	TCG	48	CTT	48	TTA	48	ACT
49	TAC	49	TCG	49	CTT	49	TTA	49	ACT
50	TAC	50	TCG	50	CTT	50	TTA	50	AGG
51	TAC	51	TCG	51	CTT	51	TTA	51	AGT
52	TAC	52	TCG	52	CTT	52	TTA	52	AGT
53	TAC	53	TCG	53	CTT	53	TTA	53	ATG
54	TAC	54	TCG	54	CTT	54	TTA	54	ATG
55	TAC	55	TCG	55	CTT	55	TTA	55	ATG
56	TAC	56	TCG	56	CTT	56	TTA	56	ATT
57	TAC	57	TCG	57	CTT	57	TTA	57	ATT
58	TAC	58	TCG	58	CTT	58	TTA	58	CAG
59	TAC	59	TCG	59	CTT	59	TTA	59	CAT
60	TAC	60	TCG	60	GAT	60	TTA	60	CAT
61	TAC	61	TCG	61	GCG	61	TTA	61	CCG
62	TAC	62	TCG	62	GCT	62	TTA	62	CCG
63	TAC	63	TCG	63	GCT	63	TTA	63	CCG
64	TAC	64	TCG	64	GTG	64	TTA	64	CCG
65	TAC	65	TCG	65	GTT	65	TTA	65	CCG
66	TAC	66	TCG	66	TAA	66	TTA	66	CCG
67	TAC	67	TCG	67	TAT	67	TTA	67	CTG
68	TAC	68	TCG	68	TAT	68	TTA	68	GAG
69	TAC	69	TCG	69	TCT	69	TTA	69	GCG
70	TAC	70	TCG	70	TCT	70	TTA	70	GCG
71	TAC	71	TCG	71	TCT	71	TTA	71	GCT
72	TAC	72	TCG	72	TCT	72	TTA	72	GGG
73	TAC	73	TCG	73	TCT	73	TTA	73	GGG
74	TAC	74	TCG	74	TCT	74	TTA	74	GTG
75	TAC	75	TCG	75	TGG	75	TTA	75	GTT
76	TAC	76	TCG	76	TGG	76	TTA	76	GTT
77	TAC	77	TCT	77	TGT	77	TTA	77	TAG
78	TAC	78	TCT	78	TGT	78	TTA	78	TAT
79	TAC	79	TCT	79	TGT	79	TTA	79	TAT
80	TAG	80	TGC	80	TTG	80	TTA	80	TAT
81	TAG	81	TGT	81	TTG	81	TTA	81	TCT
82	TAT	82	TGT	82	TTG	82	TTA	82	TCT
83	TCG	83	TGT	83	TTG	83	TTG	83	TCT
84	TCT	84	TTG	84	TTG	84	TTG	84	TGG
85	TGG	85	TTG	85	TTG	85	TTG	85	TGT
86	TGT	86	TTG	86	TTG	86	TTG	86	TTG
87	TGT	87	TTG	87	TTT	87	TTG	87	TTG
88	TTG	88	TTG	88	TTT	88	TTG	88	TTG
89	TTG	89	TTG	89	TTT	89	TTG	89	TTT
90	TTG	90	TTT	90	TTT	90	TTT	90	TTT
91	TTT	91	TTT	91	TTT	91	TTT	91	TTT
		92	TTT			92	TTT		
						93	TTT		

Table S3. Screening results of two-step PCR approach with non-overlapping primers for NNK-based site-saturation mutagenesis.

	Y51X		S72X		L75X		L437X		T438X
1	AAG	1	AAG	1	AAG	1	AAG	1	AAG
2	AAG	2	AAG	2	AAG	2	AAG	2	AAT
3	AAT	3	AAG	3	AAT	3	AAG	3	AAT
4	AAT	4	AAG	4	AAT	4	AAT	4	AAT
5	AAT	5	AAT	5	AAT	5	AAT	5	AAT
6	ACG	6	AAT	6	AAT	6	AAT	6	AAT
7	AGG	7	AAT	7	AAT	7	AAT	7	ACG
8	AGG	8	AAT	8	ACT	8	AAT	8	ACG
9	AGG	9	AAT	9	AGG	9	AAT	9	ACG
10	AGG	10	AAT	10	AGG	10	AAT	10	ACG
11	AGT	11	ACG	11	AGG	11	AAT	11	ACG
12	AGT	12	AGG	12	AGT	12	ACT	12	ACG
13	ATG	13	AGT	13	AGT	13	ACT	13	ACG
14	ATG	14	AGT	14	AGT	14	ACT	14	ACG
15	ATG	15	AGT	15	AGT	15	ACT	15	ACG
16	ATG	16	ATG	16	ATG	16	ACT	16	ACT
17	ATT	17	ATG	17	ATG	17	ACT	17	ACT
18	ATT	18	ATG	18	ATG	18	ATG	18	ACT
19	CAG	19	ATG	19	ATT	19	ATG	19	ACT
20	CAG	20	ATT	20	CAT	20	ATG	20	ACT
21	CAG	21	ATT	21	CCG	21	ATG	21	ACT
22	CAG	22	ATT	22	CCT	22	ATT	22	ACT
23	CAG	23	ATT	23	CGG	23	ATT	23	ACT
24	CCG	24	ATT	24	CGT	24	ATT	24	AGG
25	CCT	25	CAG	25	CGT	25	ATT	25	AGT
26	CCT	26	CAT	26	CTG	26	ATT	26	AGT
27	CGG	27	CCG	27	CTG	27	CAG	27	AGT
28	CGT	28	CCT	28	CTT	28	CAG	28	ATG
29	CGT	29	CGT	29	CTT	29	CAT	29	ATG
30	CGT	30	CGT	30	GAG	30	CAT	30	ATT
31	CGT	31	CTG	31	GAT	31	CAT	31	ATT
32	CTT	32	CTG	32	GAT	32	CAT	32	ATT
33	GAG	33	CTT	33	GCG	33	CAT	33	ATT
34	GAG	34	GAG	34	GCG	34	CAT	34	CAG
35	GAG	35	GAG	35	GCT	35	CAT	35	CAG
36	GAG	36	GAG	36	GCT	36	CCG	36	CAG
37	GAG	37	GAT	37	GCT	37	CCG	37	CAG
38	GAG	38	GAT	38	GGG	38	CCT	38	CAT
39	GAG	39	GAT	39	GGG	39	CCT	39	CAT
40	GAT	40	GCT	40	GGG	40	CCT	40	CAT
41	GCG	41	GTC	41	GGT	41	CCT	41	CAT
42	GCT	42	GTG	42	GGT	42	CCT	42	CAT
43	GGG	43	GTT	43	GGT	43	CCT	43	CAT
44	GGG	44	GTT	44	GGT	44	CCT	44	CAT
45	GGG	45	GTT	45	GTG	45	CGG	45	CAT

46	GGG	46	GTT	46	GTG	46	CGG	46	CCG
47	GGG	47	GTT	47	GTG	47	CGT	47	CCT
48	GGG	48	GTT	48	GTG	48	CTG	48	CCT
49	GGT	49	TAG	49	GTG	49	CTT	49	CCT
50	GGT	50	TAG	50	GTT	50	CTT	50	CGG
51	GGT	51	TAG	51	GTT	51	CTT	51	CGT
52	GGT	52	TAG	52	GTT	52	CTT	52	CGT
53	GTG	53	TAT	53	GTT	53	CTT	53	CGT
54	GTG	54	TAT	54	GTT	54	CTT	54	CTG
55	GTG	55	TAT	55	GTT	55	GAG	55	CTT
56	GTG	56	TAT	56	TAG	56	GAT	56	CTT
57	GTG	57	TAT	57	TAG	57	GAT	57	GAG
58	GTG	58	TAT	58	TAT	58	GCG	58	GAT
59	GTT	59	TAT	59	TAT	59	GCG	59	GAT
60	GTT	60	TAT	60	TAT	60	GCG	60	GAT
61	TAC	61	TAT	61	TAT	61	GCT	61	GCG
62	TAC	62	TAT	62	TAT	62	GCT	62	GCG
63	TAC	63	TAT	63	TAT	63	GCT	63	GCG
64	TAC	64	TCG	64	TCG	64	GCT	64	GCG
65	TAG	65	TCG	65	TCT	65	GCT	65	GCT
66	TAG	66	TCG	66	TCT	66	GGG	66	GCT
67	TAG	67	TCG	67	TCT	67	GGT	67	GCT
68	TAT	68	TCG	68	TCT	68	GGT	68	GCT
69	TAT	69	TCT	69	TCT	69	GTA	69	GTT
70	TAT	70	TGG	70	TCT	70	GTG	70	GTT
71	TAT	71	TGT	71	TGG	71	TAG	71	TAG
72	TCG	72	TTG	72	TGG	72	TAG	72	TAG
73	TCT	73	TTG	73	TGG	73	TAT	73	TAT
74	TGG	74	TTG	74	TGG	74	TAT	74	TAT
75	TGG	75	TTG	75	TGG	75	TAT	75	TAT
76	TGG	76	TTG	76	TGG	76	TAT	76	TCG
77	TGG	77	TTG	77	TGT	77	TCG	77	TCT
78	TGG	78	TTG	78	TGT	78	TCT	78	TCT
79	TGT	79	TTG	79	TGT	79	TCT	79	TCT
80	TGT	80	TTT	80	TGT	80	TCT	80	TCT
81	TGT	81	TTT	81	TGT	81	TCT	81	TCT
82	TGT	82	TTT	82	TGT	82	TGG	82	TCT
83	TGT	83	TTT	83	TGT	83	TGT	83	TCT
84	TGT	84	TTT	84	TTG	84	TGT	84	TCT
85	TTG	85	TTT	85	TTG	85	TTA	85	TGG
86	TTG	86	TTT	86	TTG	86	TTA	86	TGT
87	TTG	87	TTT	87	TTG	87	TTA	87	TGT
88	TTG	88	TTT	88	TTG	88	TTA	88	TGT
89	TTT	89	TTT	89	TTT	89	TTA	89	TGT
90	TTT	90	TTT	90	TTT	90	TTG	90	TTG
91	TTT	91	TTT	91	TTT	91	TTT	91	TTG
		92	TTT	92	TTT	92	TTT		
						93	TTT		
